# Translating efficacy of liver transplantation in liver-limited metastatic colorectal cancer into clinical practice: the TransMet trial

**DOI:** 10.1016/j.esmoop.2024.103669

**Published:** 2024-08-20

**Authors:** M.M. Germani, N. Raschzok, V. Heinemann, D.P. Modest

**Affiliations:** 1 Department of Translational Research and New Technologies in Medicine, University of Pisa, Pisa; 2 Unit of Medical Oncology 2, Azienda Ospedaliero-Universitaria Pisana, Pisa, Italy; 3 Department of Surgery, Campus Charité Mitte/Campus Virchow-Klinikum, Charité – Universitätsmedizin Berlin Berlin; 4 Berlin Institute of Health at Charité – Universitätsmedizin Berlin, BIH Biomedical Innovation Academy, BIH Charité Clinician Scientist Program, Berlin; 5 Department of Medical Oncology and Comprehensive Cancer Center Munich, Ludwig-Maximilian-University (LMU) of Munich, Munich; 6 Medical Department, Division of Hematology, Oncology and Tumor Immunology, Charitè Universitaetsmedizin Berlin, Berlin, Germany

**Keywords:** liver transplantation, liver-limited metastatic colorectal cancer

## Abstract

Pioneer studies suggested that liver transplantation (LT) has the potential to provide long-term survival in patients with liver-limited metastatic colorectal cancer (mCRC) not amenable for surgery of metastases. Evidence, however, was limited to single-arm studies with few patients enrolled and suboptimal selection criteria, with concerns over access to organ availability overcoming the potential efficacy of LT in this setting. Recently, 5-year survival rates with chemotherapy followed by LT (73%) compared with chemotherapy alone (9%) have been demonstrated by the randomized TransMet trial, enrolling 94 definitively unresectable strictly selected liver-limited mCRC patients. These findings should now prompt clinical oncologists to reconsider LT as a valuable option for unresectable liver-limited mCRC patients meeting TransMet criteria, and transplantation agencies to adapt their policies of access to organ donation.

## Highlights

• Liver transplantation (LT) resulted in longer overall survival in patients with definitively unresectable liver-limited mCRC.
• A centralized approach with a board of experts has the potential to standardize LT procedures in the real-world practice.
• Marginal grafts and living donors can address the current shortage of livers to meet the organ demand for mCRC.
• Whether mCRC patients should be prioritized in the waiting list for LT is a key ethical dilemma for transplantation agencies.

Expectations of long-term survival in metastatic colorectal cancer (mCRC) are mainly restricted to patients amenable for radical resection of secondary lesions and primary tumor.^1^ This is mostly the case in patients with metastatic spread limited to the liver. For all other patients, long-term survivorship is realistically attainable only by immunotherapy in tumours with deficient mismatch repair/microsatellite instability-high (dMMR/MSI-H) accounting, however, for only 5% of mCRC diagnoses.^1^ Therefore, when the disease is liver limited, but not initially resectable in proficient mismatch repair (pMMR) tumours, the joint effort of multidisciplinary teams including oncologists, surgeons, radiotherapists, and interventional radiologists is necessary to achieve complete eradication of liver metastasis.^1^ In these patients, intensified cytotoxic regimens combined with monoclonal antibodies and increasingly complex interventional procedures can achieve conversion to resectability at a rate up to 40%-50%.^2^ By contrast, if metastatic disease is not amenable for complete eradication, the outcome is almost invariably fatal.

When surgery is not feasible because of insufficient remnant of healthy tissue or involvement of critical structures, liver transplantation (LT) can be a solution.^3^ This was the pioneering approach in two single-arm Scandinvian trials (SECA-I and II), enrolling 21 and 15 patients, respectively. Besides feasibility, the most important evidence collected was the improved 5-year overall survival (OS) rate in SECA-II (83%), compared with SECA-I (44%), due to stricter selection criteria applied in the SECA-II trial (Table 1).^4^^,^^5^ Despite selected transplanted patients exceeding the median OS expected for initially unresectable patients (<5 years),^6^^,^^7^ these findings met legitimate concerns on the small sample size, perioperative morbidities, and social costs associated with parsimonious allocation of transplantable organs. Also, it appeared that even a drastic intervention like transplantation was far from curing patients, with roughly two-thirds relapsing within 2 years after the procedure.^4^^,^^5^

**Table 1:** Eligibility criteria in main clinical trials on liver transplantation in liver-limited CRC

	SECA-I	SECA-II	TransMet
Confirmation of no extrahepatic disease	CT scan, FDG–PET–CT and bone scan	CT scan and FDG–PET–CT	CT scan and FDG–PET–CT
Assessment of unresectability	National, centralized at one centre	National, centralized at one centre	International, centralized
Age	Not specified	Not specified	18-65 years
Primary tumour	Resected	Resected	Resected
ECOG-PS	0/1	0/1	0/1
Line of chemotherapy	Not specified	Not specified	≤3
Response to CT (RECIST criteria)	Not specified	• At least 10%. • If >20 lesions (see below) at least 30%. • If <10% eligible if 20% response achieved after TACE or ^90^Y-spheres	At least stable disease lasting ≥3 months
Radiological criteria	Not specified	• No lesion >10 cm before the start of CT • If >20 lesions all <5 cm	Not specified
Molecular criteria	Not specified	Not specified	*BRAF* wild-type
Biochemical criteria	Not specified	Not specified	CEA level <80 ng/ml or ≥50% decrease from baseline
Other criteria	—	At least 1-year time span from CRC diagnosis and date of being listed on the transplantation list	—

CEA, carcinoembryonic antigen; CT, computed tomography; ECOG-PS, Eastern Cooperative Group performance status; FDG-PET, [^18^F]2-fluoro-2-deoxy-D-glucose–positron emission tomography; (m)CRC, (metastatic) colorectal cancer; TACE, transarterial chemoembolization.

This perception of LT in mCRC has recently been overturned at the 2024 Annual ASCO Meeting by Adam et al.,^8^ that presented the outcomes of the TransMet trial, the first randomized study comparing chemotherapy followed by LT with chemotherapy in definitively unresectable liver-limited mCRC patients. Despite the challenging clinical scenario, 157 patients were submitted to a centralized validation committee and 94 randomized in 4.5 years. After a median follow-up of 59 months, chemotherapy followed by LT was superior to chemotherapy alone, with a 5-year OS rate of 57% versus 13% (*P* = 0.0003) in the intention-to-treat population and even a greater advantage in the per-protocol population (73% versus 9%, *P* < 0.0001).^8^ The recurrence rate was 72%.^8^ Nonetheless, consistent with previous observations,^4^^,^^5^ relapses were mostly confined to one organ (88%), especially the lung (54%), with 12 out of 26 relapsed patients (46%) undergoing surgery or ablation.^8^ At the end of the observation period, 15 had no evidence of disease (NED) in the LT arm (42%) versus 1 (3%) in the chemotherapy arm.^8^ Importantly, the well-known concerns on long-term sequalae of LT were blown out by quality of life (QoL) and post-LT chemotherapy data, suggesting that transplanted patients did not experience greater global heath score deterioration, with apparently no meaningful impact on feasibility and tolerability of post-LT chemotherapy.^9^

The most obvious consequence of the TransMet trial should be a meltdown of scepticism of clinical oncologists. Despite the genuine concerns on the availability of suitable liver allografts and relapses, the unprecedented 5-year survival rate achieved after LT in a randomized setting of proper dimension, consistent with previous findings from the SECA-II trial,^5^ cannot be ignored and LT still labelled as ‘investigational’ in international guidelines.^1^ Whenever a liver-limited mCRC patient cannot be resected, LT should be considered, as long as the patient is adequately selected.

Despite the potential innovation of the study, its applicability presents some limitations. The first bottleneck of selection is represented by biomarkers of disease aggressiveness. Far before the TransMet trial, there was a shared awareness on clinical and molecular features warranting against LT, which were a primary tumour in place, unresponsiveness to chemotherapy, *BRAFV600E* mutation, a biomarker of disease aggressiveness, and a dMMR/MSI-H *status*, that grants long-term survival with immunotherapy.^3^ Nonetheless, other factors do not share the same agreement, especially regarding the dimension of liver lesions and the biological behaviour, included in the Oslo Score, resulting from the Scandinavian experience,^10^ and in guidelines based on international *consensus* (Table 1).^3^ Regardless of the selection criteria, a key lesson from the TransMet trial is that a centre-level approach has a high risk (40%) of failing to identify optimal LT candidates, even within a study protocol with explicit selection criteria.^11^ Therefore, a centralized approach implementing an expert committee seems essential for bringing standardization of procedures and initial verification of candidates in a real-world scenario.

The second bottleneck for LT is definitive unresectability of metastases. Unfortunately, what is meant by ‘unresectable’ can still be a grey area and even in assessments within highly experienced liver surgeons, mixed perspectives occur in a substantial subset of patients.^2^^,^^12^ Still, a centralized review may help to set the bar of technical (un)resectability compared with a centre-level approach, and this occurred in 8% of resectable cases submitted to the TransMet validation committee. Furthermore, an additional 15% of apparently unresectable patients later underwent surgery in the control arm of the study.^8^ It remains to be determined whether resection was feasible only through complex procedures, such as portal vein embolization (PVE) or associated liver partition with portal vein ligation for staged hepatectomy (ALPPS), which are associated with high perioperative morbidity and potentially worse outcomes compared with LT.^13^ Additionally, since relapses after LT are mainly in the lung but may still be resected,^8^ it could be speculated that the presence of resectable synchronous lung metastases should not absolutely contraindicate LT. Some trials are assessing if LT can be proposed in this setting.^14^

**Figure 1:**
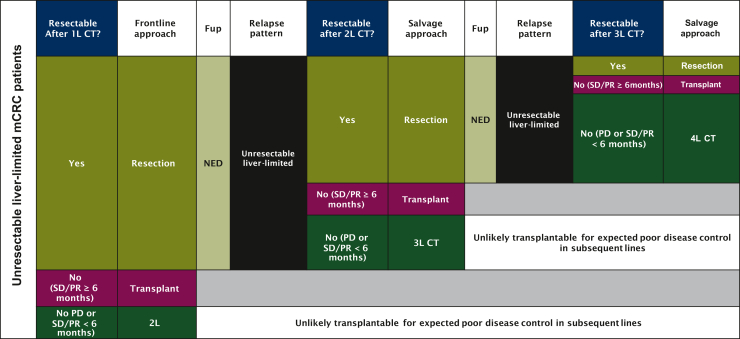
**Real-world scenarios of application of liver transplantation in liver-limited mCRC.** 1L, first-line; 2L, second-line; 3L, third-line; 4L, fourth-line; CT, chemotherapy; Fup, follow-up; mCRC, metastatic colorectal cancer; NED, no evidence of disease; PD, progressive disease; PR, partial response; QoL, quality of life; SD, stable disease.

A third crucial point could be the selection process: may it work on an individual-based level, its sustainability on a community level is far from clear. A rough estimate coming from 12 academic Italian and German trials including 4756 mCRC patients suggests that only 190 patients (4%) may be eligible to LT in first line using TransMet and Oslo criteria.^15^ This may not be the only potential scenario, however, with a potential bulk of liver-limited patients relapsing after initial hepatic surgery (Figure 1). Although these numbers do not tell the entire story on a population level, they at least uncover the dimensions of the problem. Even when all oncological concerns have been addressed, however, the lack of suitable liver allografts will limit the widespread clinical adoption. Comprehensive data from the Eurotransplant registry similarly showed that between 2006 and 2015, 32% of patients were removed from the waiting list as they became too sick to receive a transplant or died on the waiting list.^16^

Although the decay of hepatitis C virus (HCV) decompensated cirrhosis in the direct-acting antiviral era has partially relieved the pressure of liver donor shortage, this may not be sufficient to address the potential *surplus* of grafts needed to absorb the new demand of LT.^17^ So called *marginal* grafts from donors fulfilling criteria that have been shown to confer an increased risk for poor graft and patient survival can be successfully transplanted after careful selection, and in the context of advancements in machine perfusion technologies which enable improved preservation and allow for quality assessment and potentially rehabilitation of such grafts.^18^^,^^19^ Moreover, the potential number of donors could be further increased through a more liberal use of living donor liver transplantation (LDLT). LDLT, however, can be tackled by meaningful morbidities and long-term health and social costs of the donor, depending on the graft needed to match the donor and recipient anatomy.^20^ Anyway, from an ethical standpoint, while the cost of LDLT may be outweighed by the benefit of achieving cure for non-oncological indications, especially in children, this may not be the case in the majority of liver-limited mCRC patients, who experience disease recurrence after 72% of LT. Also, while the 73% 5-year survival rate is encouraging in unresectable pMMR/MSS mCRC patients, cost-effectiveness may be questioned if the same organ may provide decades of life expectancy in a non-oncological scenario.

Although the shortage of liver donors may be less demanding in the upcoming years, some ethical dilemmas still remain. One key aspect driving early access to the donor pool in TransMet was that the validation committee had the power to prioritize liver allocation for transplantable mCRC patients.^8^^,^^11^ From an oncological standpoint, this appears reasonable due to the narrow window of opportunity for LT, given the high rate of disease progression while on the waiting list (21% in the bimonthly window of TransMet). Whether a commission should hold such power in a real-world scenario, however, might be debatable. The ethical issues behind this approach must be faced in synergism with transplantation agencies.

In conclusion, data are now solid enough to shift the paradigm of LT also in definitively unresectable liver-limited mCRC patients. The TransMet trial has also opened the path towards a model that can make this new paradigm work, but also poses substantial logistical hurdles and ethical dilemmas that healthcare providers and policy makers have to address to translate this model into real-world practice.
